# Notes and News

**Published:** 1899-11-25

**Authors:** 


### Notes and News.

(Collected and Communicated.)

Lord Curzon's visit to the Plague Camps and Hospitals in Poona has had a very good effect amongst the native population.

Three cases of plague, all of which terminated fatally, are announced on board an Austrian Lloyd steamer on her voyage from Brazil to Trieste. No further spread of the disease is reported.

Alderman Treloar is making his annual appeal for Christmas dinners and hampers for the crippled children of the poor in London. Last year 4,324 hampers, we are told, were sent to children who could not attend the banquet provided in the Guildhall. Subscriptions should be addressed to Alderman Treloar, Ludgate Hill.

The Queen has granted a Royal Charter of Incorporation to the British Home for Incurables at Streatham, under the name and designation of the "British Home and Hospital for Incurables." The Lady Mayoress, accompanied, it is hoped, by the Lord Mayor and the Sheriffs, will pay a visit to the institution on the afternoon of Monday, December 4th, for the purpose of unveiling the new east window in the chapel, and for opening the sale of work made by the patients for their own benefit.

During the last two months there has been a very large increase of typhoid fever in the metropolitan area, 1,526 notifications having been received in the ten weeks ending November 4th, compared with 937 during the corresponding weeks the year before. The disease appears to be increasing, especially in the eastern, north-eastern, and south-eastern districts. All the beds at the disposal of the Metropolitan Asylums Board for the reception of such cases have been filled, and many cases have been sent to general hospitals, but there still remains a considerable number of cases which are awaiting removal. The Metropolitan Hospital has placed unfurnished two wards at the disposal of the Board, and these will be immediately fitted up, the Local Government Board having already given their sanction to the arrangement. Nevertheless, even with this help, there are not as yet nearly sufficient beds available for all the cases which it would be desirable to remove to hospital.

A new male infirmary has been opened by the Kingston Board of Guardians, to accommodate 132 inmates. The building consists of three floors, on each of which are five wards, a nurses' duty-room, and the usual offices. Adjoining is a home for the nurses. The cost has been £20,000. Mr. W. H. Hope, of Kingston, is the architect.

A festival dinner in connection with the Victoria Hospital for Children was to have been held at the Hotel Cecil on Wednesday, November 8th, under the presidency of Field-Marshal Lord Roberts, V.C., but has been postponed, on account, we understand, of the trouble in the Transvaal, which has rendered the present an inauspicious time for the appeal which takes such a prominent place in such proceedings.

Not everyone can give as much in money as they would wish to assist sufferers from the war in the Transvaal, but there will be many who can render substantial service by sending some suitable garments for the use of the refugees. Friends in England are now learning to what plight many of those are put who have had to flee away as best they might, taking very few worldly goods with them. The shipping companies have undertaken to convey clothes at half rates, and all contributions may be sent to Messrs. Hayter and Hayter, George Yard, Upper Thames Street, from whence they will be conveyed to the Cape through the agency of the Mansion House Fund, and at its expense.

The records kept in Germany in regard to sick insurance, which is now compulsory upon workmen, are beginning to show how serious a strain sickness produces upon a country. It appears that nearly eight million persons in Germany subscribe to the Krankenkassen—that is, ensure against sickness, and that in 1898 one-third of these reported illness of one sort or another, the average duration of sickness being 17 days. Now, if we reckon wages at only 2s. a day, this comes to a loss of more than four and a half million pounds a year in wages alone, besides what has somehow to be provided for food, physic, and maintenance. This, of course, is among working people during the working period, which is, presumably, the most healthy period of their lives.

Davos-Platz has added a funicular railway to its other attractions. It is about 2,200 ft. long, starting behind the Kurhaus and going up to the Schatzalp. The motive power is electricity. An electric tramway is also going to be inaugurated between Davos-Platz and Davos-Dorf. Funiculars and electric trams are, in our opinion, a great advantage to health resorts, for by their aid the field in which exercise can be taken is much widened, and life is thereby made more interesting. For tobogganing purposes, however, a funicular is not all gain. For those who are fairly strong the somewhat weary climb is quite as useful, and perhaps more health-giving, than the delirious shoot down-hill; while there is always the probability to be reckoned with that the facilities offered by the funicular may tempt many to use the toboggan who are by no means fit for the rapid rush through the air. Pleasant and exciting as this may be, it involves most of the well-recognised evils of exposure to a high wind.

